# The General Medical Council

**Published:** 1898-12-10

**Authors:** 


### THE GENERAL MEDICAL COUNCIL.

Our report of the proceedings of the General Medical Council, last week, was carried down to the adjournment on Tuesday evening, November 29th.

On Wednesday, the 30th, after an explanation from Dr. Pettigrew of a statement which he had made with reference to the marks given for different subjects in the preliminary examinations of Scottish Universities, the first business was the consideration of a motion of which notice had been given by Mr. Horsley, but which was ruled by the President to be out of order, and which, therefore, was not proceeded with. A report from the President and Treasurers with regard to alterations in the Council building was read and adopted. Its recommendations were that the building should be raised by an additional story, which would afford a room for the president and additional committee rooms, and that the offer of the English branch to advance on mortgage the required sum of £3,000 should be accepted, with the thanks of the Council. It was mentioned that the contractors were prepared to complete the work in time for the meeting of Council in May, and it was arranged that advantage should be taken of the opportunity for improving the ventilation of the Council chamber itself. Consideration of the report of the committee, on a proposed revision of the Council's report on reciprocity of practice in foreign countries, was adjourned until the next session. A report from the Executive Committee, relating to a modification of a standing order on the subject of practitioners convicted of felony or misdemeanour, was received and adopted. The modified order gives power to the president to refer any such conviction, either to the Penal Cases Committee, or to the Branch Council for the part of the kingdom in which the offence was committed; in either case for report to the next session of the Council itself. A report from the Pharmacopœia Committee, stating that the work had been well received, that 20,500 copies had been sold, and that progress had been made with the addendum for India and the Colonies, was received and adopted. On an application from the Medical Defence Union, with reference to the case of an unqualified practitioner, it was determined to consider this and certain other items of business *in camerâ*; and, on the readmission of strangers, it was announced that a resolution had been proposed by Mr. Horsley, and seconded by Mr. Brown, that members of the profession visiting the session of the Council shall be furnished with a copy of the daily programme of business, and that the motion had been negatived. The explanation of this was understood to be that the programme may sometimes contain complaints against individuals or other matter which might be treated as libellous, but which, when confined to members of Council, has a character of privilege which it would lose if it were more generally circulated.

On Thursday, December 1st, the first business was the consideration of the report of a Committee on the Employment of Unqualified Assistants; a report which is adopted, and is discussed in another column. A "Medical Ordinance," from the Colony of Trinidad, was received from the Colonial Office, and was found to contain more stringent regulations from the prevention of unqualified practice than exist in any other part of the Empire. A resolution was passed signifying full approval of these regulations on the part of the Council.. It was announced that the Local Government Board was preparing a scheme to meet difficulties in the way of instructing students in vaccination, which might be expected to arise from the abandonment of the present Vaccination Stations; and the further consideration of this question was therefore deferred. The Committee of Medical Aid Associations presented a third interim report, setting forth that negotiations with the authorities of the chief trade societies were proceeding favourably. A resolution was passed approving of the formation of a Sinking Fund for the repayment in fifty years of the money, in all £25,000, which has been advanced by the English branch to the Council, but the details of the scheme were left to be determined hereafter. It was determined to appoint an inspector for certain examinations in Ireland, in the room of Dr. Coupland, who had resigned office on his appointment as a Commissioner in Lunacy; and authority was given to the President for this purpose. A letter from the Royal College of Surgeons of Ireland, with regard to the recognition of boys' schools as places for the instruction of medical students in biology, physics, chemistry, and practical chemistry, was referred to the Education Committee for report to the next session of the Council. The Executive Committee was directed to report upon the arrangements for facilitating the access of members of the Council to documents which are the property of the Council, with due regard to the safety of such documents, and to the rights of the Council as a whole. The Committee on Fines under the Medical Acts, which, in the present state of the law, if inflicted in the metropolitan district, are not paid to the Council, but to the Receiver of the Police, reported that they had been advised to seek an interview with the Secretary of State upon the subject, and received authority to do so. A motion of which notice had been given by Mr. Brown, affirming that it would be expedient to confer upon registered practitioners in England the power of returning an additional member to the Council, was, by the permission of the Council, withdrawn. Mr. Brown called attention to an alleged infringement of the Medical Acts by persons who gave certificates of proficiency to opticians and other tradesmen, and the matter was referred to the Executive Committee. A motion that the Registrar, "in all cases" in which an unregistered person charged with an offence described himself as a medical practitioner, should write to the presiding official of the tribunal concerned, stating that the accused was not a registered practitioner, was lost.

On a report as to the facts by the Dental Committee, the Council decided to take no action with regard to charges brought against Edward Browning, Charles John Dalton, and Henry Rowton; and, on another report from the members of the same committee, the Council authorised the issue of certain notices with regard to the covering of unqualified dental practitioners, and especially with regard to the administration of anæsthetics for such persons by duly qualified medical men. It was pointed out to the Irish Branch Council that the action which they had resolved to take, by keeping a branch dental register, was beyond the powers conferred upon them by the Dentists' Act.

An application from Ernest Harold Christopher Bailey, a dentist of Ballarat, in the colony of Victoria, to be registered as a colonial dentist was considered and acceded to. The Council then adjourned.

We are informed that between the assembling of the General Medical Council on Saturday and four p.m. on Thursday, when the record was discontinued, Mr. Horsley made 73 speeches and Mr. Brown made 60, so that the profession at large, by aid of its direct representatives, can no longer be considered inarticulate.

